# Identification of Mutations in the *mrdA* Gene Encoding PBP2 That Reduce Carbapenem and Diazabicyclooctane Susceptibility of Escherichia coli Clinical Isolates with Mutations in *ftsI* (PBP3) and Which Carry *bla*_NDM-1_

**DOI:** 10.1128/mSphere.00074-19

**Published:** 2019-07-03

**Authors:** Srijan Ranjitkar, Folkert Reck, Xiaobo Ke, Qingming Zhu, Glenn McEnroe, Sara L. Lopez, Charles R. Dean

**Affiliations:** a Infectious Diseases, Novartis Institutes for BioMedical Research, Emeryville, California, USA; b Chemical Biology and Therapeutics, Novartis Institutes for BioMedical Research, Cambridge, Massachusetts, USA; Antimicrobial Development Specialists, LLC

**Keywords:** NDM-1, carbapenems, diazabicyclooctane, penicillin-binding proteins

## Abstract

Penicillin-binding proteins (PBPs) are essential for bacterial cell wall biosynthesis, and several are clinically validated antibacterial targets of β-lactam antibiotics. We identified mutations in the *mrdA* gene encoding the PBP2 protein in two Escherichia coli *bla*_NDM-1_ clinical isolates that reduce susceptibility to carbapenems and to the intrinsic antibacterial activity of a diazabicyclooctane (DBO) PBP2 and β-lactamase inhibitor. These mutations coexisted with previously described mutations in *ftsI* (encoding PBP3) that reduce susceptibility to monobactams, penicillins, and cephalosporins. Clinical exposure to β-lactams is driving the emergence of multifactorial resistance that may impact the therapeutic usefulness of existing antibacterials and novel compounds that target PBPs.

**IMPORTANCE** Emerging antibacterial resistance is a consequence of the continued use of our current antibacterial therapies, and it is limiting their utility, especially for infections caused by multidrug-resistant isolates. β-Lactams have enjoyed extensive clinical success, but their broad usage is linked to perhaps the most extensive and progressive example of resistance development for any antibacterial scaffold. In Gram-negative pathogens, this largely involves constant evolution of new β-lactamases able to degrade successive generations of this scaffold. In addition, more recently, alterations in the targets of these compounds, penicillin-binding proteins (PBPs), are being described in clinical isolates, which often also have multiple β-lactamases. This study underscores the multifactorial nature of β-lactam resistance by uncovering alterations of PBP2 that reduce susceptibility to carbapenems in E. coli clinical isolates that also have alterations of PBP3 and express the NDM-1 β-lactamase. The changes in PBP2 also reduced susceptibility to the intrinsic antibacterial activity of some diazabicyclooctane (DBO) compounds that can target PBP2. This may have implications for the development and use of the members of this relatively newer scaffold that are inhibitors of PBP2 in addition to their inhibition of serine-β-lactamases.

## Abstract (precis)

Emerging antibacterial resistance is a consequence of the continued use of our current antibacterial therapies, and it is limiting their utility, especially for infections caused by multidrug-resistant isolates. β-Lactams have enjoyed extensive clinical success, but their broad usage is linked to perhaps the most extensive and progressive example of resistance development for any antibacterial scaffold. In Gram-negative pathogens, this largely involves constant evolution of new β-lactamases able to degrade successive generations of this scaffold. In addition, more recently, alterations in the targets of these compounds, penicillin-binding proteins (PBPs), are being described in clinical isolates, which often also have multiple β-lactamases. This study underscores the multifactorial nature of β-lactam resistance by uncovering alterations of PBP2 that reduce susceptibility to carbapenems in E. coli clinical isolates that also have alterations of PBP3 and express the NDM-1 β-lactamase. The changes in PBP2 also reduced susceptibility to the intrinsic antibacterial activity of some diazabicyclooctane (DBO) compounds that can target PBP2. This may have implications for the development and use of the members of this relatively newer scaffold that are inhibitors of PBP2 in addition to their inhibition of serine-β-lactamases.

## OBSERVATION

**FIG 1:**
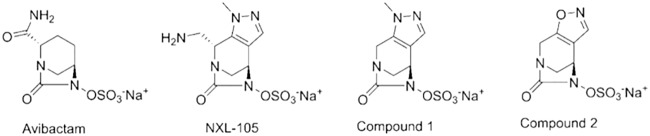
Chemical structures of diazabicyclooctane (DBO) molecules. NXL-105 is described in reference 11 (US2010092443 [12]), and compounds 1 and 2 are described in WO 02/100860 (13).

β-Lactams are a broad class of antibacterial agents that inhibit penicillin-binding proteins (PBPs) essential for transglycosylation and transpeptidation of peptidoglycan strands during bacterial cell wall biosynthesis (1). Many β-lactam antibiotics have been developed and extensively used in the clinic over the past several decades. These antibiotics have a wide range of PBP affinities and specificities, with most inhibiting multiple PBPs (2). Unfortunately, the utility of this class of antibiotics is being undermined by continuing resistance development. In particular, resistance to carbapenems, a group that has been widely used as an agent of last resort to treat severe infections caused by extended-spectrum-β-lactamase (ESBL)-expressing Enterobacteriaceae, is a growing concern (3). Clinical resistance to β-lactams in Gram-negative bacteria had primarily been attributed to the expression of plasmid-carried or chromosomal β-lactamase genes (4), with reports of specific target mutations being rare in Gram-negative pathogens. Recently, however, mutations in *ftsI* encoding insertions in PBP3 that reduce susceptibility to certain β-lactams have been identified in Escherichia coli clinical isolates (5, 6). In addition, clinical isolates and *in vitro*-selected E. coli mutants with reduced susceptibility to carbapenems showed changes in the gene encoding PBP2 (7–9). Given the continuing emergence and spread of new β-lactamases, there is interest in the development of novel β-lactamase inhibitors (BLIs) (4) and in the development of novel β-lactam mimetics that are not impacted by β-lactamases (10). One such class of BLIs and β-lactam mimetics is the diazabicyclooctane (DBO) scaffold, which was discovered by chemists at Hoechst Marion Roussel, such as NXL-104 (avibactam), NXL-105 (11) (US2010092443 [12]), and compounds 1 and 2 (WO 02/100860 [13]) (Fig. 1). DBOs are potent inhibitors of β-lactamases but were originally designed as β-lactam mimetics, and some analogs such as NXL-105 also had intrinsic antibacterial activity through inhibition of PBP2 and were viewed as potential antibacterial drugs (11). The notion that the antibacterial activity of such compounds should be considered in drug discovery has also been echoed more recently (14). Antibacterials that act mainly by inhibition of PBP2 can exhibit relatively high frequencies of selecting non-target-based resistance *in vitro* via a multiplicity of mutations affecting the stringent response, thereby reducing susceptibility to inhibition of PBP2 (15, 16). The potential for target-based resistance to DBOs is currently not well understood. We reported previously (17) that four NDM-1-expressing E. coli clinical isolates (NB27236, NB27330, NB27307, and NB27326 [Table 1]) possessed a previously characterized (5) mutation in *ftsI* encoding a YRIN insertion in PBP3 that reduces susceptibility to PBP3 inhibitors such as aztreonam (which is not degraded by NDM-1). Intriguingly, one of these isolates, NB27307, was 4-fold less susceptible to the antibacterial activity of the very potent DBO molecule NXL-105 than was NB27236 or the control E. coli strain NB27001 and 2-fold less susceptible than NB27330 (Table 1), based on broth microdilution assay according to CLSI methodology (18). Strain NB27326 was also 2- to 4-fold less susceptible than NB27236 and NB27001 (Table 1). Strains NB27307 and NB27326 were also less susceptible to the less potent DBO compounds 1 and 2 (Fig. 1) than were NB27236 and NB27330 (4- to 16-fold) and were 64-fold (compound 1) and 8-fold (compound 2) less susceptible than the control strain NB27001 (Table 1). However, strains NB27236 and NB27330 were also less susceptible to compound 1 (8- to 16-fold) than the control strain NB27001 whereas they did not show these substantial shifts in susceptibility to compound 2 (Table 1). Since the antibacterial activity of DBOs is mediated by inhibition of PBP2 (14), we asked if NB27307 and NB27326 harbored mutations in *mrdA*, which encodes PBP2. PCR amplification and sequencing of *mrdA* from all four isolates, and the control strain NB27001 (ATCC 25922), using the primers listed in Table 2, indicated that NB27307 and NB27326, the two isolates that were the most consistently less susceptible to DBOs, each had unique mutations encoding amino acid substitutions in PBP2 compared to the other isolates or to the E. coli ATCC 25922 or strain MG1655 *mrdA* reference sequences (19). These were T1718A, encoding a unique L573Q substitution for strain NB27307, and G1564A, encoding a unique V522I substitution for strain NB27326 (Table 1). NB27326 also had a V217M substitution in addition to V522I, while the two more-susceptible isolates, NB27236 and NB27330, harbored only the V217M substitution.

**TABLE 1:** Mutations identified in E. coli clinical isolates and antibiotic susceptibilities of engineered E. coli *mrdA* mutants^*a*^

E. coli strain	Source	PBP2 alteration	MIC (μg/ml) of drug:						
			NXL-105	Comp 1	Comp 2	IPM	MEM	ATM	CAZ
NB27001	ATCC 25922	None	0.008	0.5	2	0.25	≤0.06	0.125	0.25
NB27236 (FtsI_YRIN_)	JMI	V217M	0.008	4	1–2	16	>64	>64	>64
NB27330 (*bla*_CTX-M-15_ *bla*_CMY-2_ *bla*_NDM-1_, FtsI_YRIN_)	IHMA	V217M	0.016	8	4	32	>64	>64	>64
NB27307 (*bla*_NDM-1_ FtsI_YRIN_)	ATCC BAA-2471 (27)	**L573Q**	0.032	32	16	16	32	>64	>64
NB27326 (*bla*_SHV-12_ *bla*_CMY-2_ *bla*_NDM-1_ FtsI_YRIN_)	IHMA	V217M, **V522I**	0.016–0.032	32	16	4	16	>64	>64
BW25113		None	0.004	1	2	0.25	0.032	0.125	0.25
BW25113-CDK0001	This study	L573Q	0.016	4	16	0.5	0.125	0.125	0.25
BW25113-CDK0004	This study	V522I	0.004	4	16	0.5	0.06	0.125	0.25

a Abbreviations: ATM, aztreonam; CAZ, ceftazidime; IPM, imipenem; MEM, meropenem; ATCC, American Type Culture Collection; IHMA, International Health Management Associates; Comp, compound. IPM, MEM, ATM, and CAZ were obtained from commercial sources. DBOs were synthesized at Novartis. MIC data were generated according to CLSI methodology (18). Unique amino acid substitutions in PBP2 are indicated in bold. Strain numbers are Novartis internal numbering. Strain NB27330 was isolated from the colon of a patient in India. NB27307 is a respiratory isolate from Pakistan, and NB27326 is an isolate from peritoneal fluid in India. No additional information is available for strain NB27236.

**TABLE 2:** Oligonucleotide primers used in this study

Primer purpose and name	Sequence (5′–3′)
*mrdA* (PBP2) sequencing^*a*^	
SR176	CATCACCACCAACCATCCTT
SR177	CCGTGCAGCACATCTTCATA
SR178	TGACGATATTGCTGCATTCC
SR179	GGTTCACCAGCGGTGTATTC
SR180	TGGTTTCCACGCCTAGTTATG
SR181	AGGTTTCGTTCGCTTTCAGA
SR182	CCGAATGGATGGGTAAATTC
SR183	TGTGGGATCGAGATGGACTT
Gene manipulations and diagnostic PCR	
SR200	TTGACGGTATCTCCAGCAAA
SR201	GCTAAGGCCAGAGAGGAACA
SR202	ACACCATTCCGGTTGGTATC
SR203	TACGCTCCATCATGCCAATA

a The *mrdA* gene was amplified from clinical isolates for sequencing in segments using primer pairs SR176/SR177, SR178/SR179, SR180/SR181, SR182/SR183, and SR176/SR183.

To establish whether the two unique alterations reduced susceptibility to DBOs or β-lactams that act in part by inhibition of PBP2, each alteration was introduced individually into the susceptible E. coli laboratory strain BW25113 by recombination as previously described (20). Briefly, linear DNA fragments encompassing the appropriate region of *mrdA* were amplified from E. coli clinical isolates NB27307 and NB27326 using primers SR200 and SR201 (Table 2). There were three silent *mrdA* mutations in NB27326, and these were present on the PCR fragment from this isolate. The fragments were then individually transformed into electrocompetent E. coli BW25113 cells harboring the pKD46 helper plasmid (20). Mutants were selected on individual LB agar plates (tryptone, 10 g/liter; yeast extract, 5 g/liter; NaCl, 10 g/liter; agar, 1.5%) containing 0.25% (wt/vol) arabinose and 0.25 μg/ml imipenem at 37°C. The introduced missense mutations were confirmed on the genome for BW25113-CDK0001 and BW25113-CDK0004 (Table 1) by PCR and sequencing of the *mrdA* gene using primers SR202 and SR203 (Table 2). The three silent mutations within *mrdA* from clinical isolate NB27326 were also introduced into *mrdA* of BW25113-CDK0004. Whole-genome sequencing of BW25113-CDK0001 and BW25113-CDK0004 using previously described methodology (21) confirmed that only the intended mutations were introduced on the genome. These alterations caused a modest 2- to 4-fold decrease in susceptibility to imipenem and meropenem, which act in part by inhibition of PBP2 (7) (BW25113-CDK0001 [L573Q] and BW25113-CDK0004 [V522I] [Table 1]). Susceptibility to aztreonam and ceftazidime, which do not significantly engage PBP2 (2, 22), was not affected by either alteration.

BW25113-CDK0001 also exhibited a 4-fold decrease in susceptibility to NXL-105 (11) (Table 1). No shift in NXL-105 MIC was mediated by the V522I alteration alone (BW25113-CDK0004); however, the possibility that V522I might cause a more subtle shift (lower than 2-fold) in this strain background was not tested by growth curve analysis. We speculated that less potent but structurally related DBOs such as compounds 1 and 2 (Fig. 1) might reveal a more consistent effect of both the L573Q and V522I alterations. Indeed, the activity of both compounds was decreased (4- to 8-fold, respectively) for BW25113-CDK0001 (L573Q) and BW25113-CDK0004 (V522I) (Table 1). As mentioned above, isolates NB27236 and NB27330, which harbored only the V217M alteration, were less susceptible to compound 1 than NB27001, suggesting that this change may differentially affect susceptibility to different DBO molecules, but this remains to be confirmed. It should be noted that we did not determine the PBP binding profiles for compounds 1 and 2 and therefore do not know if they differ. Nonetheless, it is possible that an additional contribution of the V217M alteration that was not tested here together with V522I in mutant BW25113-CDK0004 could be necessary to see a shift in NXL-105 susceptibility in this mutant. Lastly, both BW25113-CDK0001 and BW25113-CDK0004 were 4-fold less susceptible to the non-DBO PBP2 inhibitor amdinocillin (MIC of 1 μg/ml for both compared to 0.25 μg/ml for BW25113 or NB27001). The L573Q and related L573V substitutions were previously implicated in reducing susceptibility to amdinocillin but were only tested along with other PBP2 alterations (9, 23). Overall, our data suggest that the amino acid substitutions identified in this study affect the intrinsic antibacterial activity of these DBO molecules, but it is unlikely that they would interfere with inhibition of β-lactamases by DBOs.

The PBP2 L573Q substitution identified here in the *bla*_NDM-1_-containing E. coli clinical isolate NB27307 occurs at the same position as a previously described L573V substitution associated with decreased susceptibility to imipenem (9) and is immediately adjacent to an M574I substitution previously associated with reduced susceptibility to carbapenems (7, 23). Furthermore, an L573Q substitution emerged in E. coli during *in vitro* serial passaging studies using ertapenem (8). However, the V522I substitution identified here in the *bla*_NDM-1_-containing E. coli clinical isolate NB27326 has not, to our knowledge, been previously associated with resistance.

A BLAST search of publicly available NCBI sequences using the PBP2 L573Q variant found one E. coli genome (NZ_CP021879.1; strain AR_0137) encoding the identical PBP2 protein. E. coli strain AR_0137 is an antibiotic-resistant carbapenemase-producing strain that is part of the FDA-CDC resistant bacteria panel (https://www.ncbi.nlm.nih.gov/bioproject/294416). A similar BLAST search using only the truncated sequence KIAERLRDHKQMTAFAPYNNPQVA, encompassing L573Q, yielded the same single hit. Searching publicly available sequences using the PBP2 V522I (single) variant sequence yielded 17 E. coli genomes encoding the identical protein (GenBank accession numbers AXN84639.1, TBI06625.1, RKP81685.1, AWR48380.1, OXK11063.1, RWS74779.1, KZJ44915.1, ROK48460.1, AUY30260.1, OCO29799.1, QAY43423.1, QBC12101.1, KDG76004.1, AXP27762.1, AYL12293.1, AWA17983.1, and VCY82512.1). These strains were mainly multidrug-resistant (MDR) and carbapenem-resistant/carbapenemase-producing clinical isolates, with RWS74779.1 having been isolated from water. This suggests that the V5221 substitution in PBP2 could be emerging among MDR isolates. Searching with the PBP2 V217M V522I double variant sequence dropped the number of hits to two (GenBank accession numbers KSY14719.1 and PSF33251.1). One of these, strain 50673720, was a carbapenemase-producing clinical isolate from Norway (24).

As mentioned above, it is well established that Gram-negative bacteria can become less susceptible to PBP2 inhibition at high frequency through a multiplicity of nontarget mutations related to the stringent response (15, 16). PBP2 target mutations affecting DBO activity, being more rare, may be difficult to identify using standard *in vitro* selection experiments, given the high background of stringent response mutants. Here, we noticed a trend suggesting differential susceptibility of clinical isolates and confirmed the presence of PBP2 alterations affecting DBO susceptibility. To our knowledge, this is the first report of alterations in PBP2 that reduce susceptibility to the intrinsic antibacterial activity of a DBO molecule. Antibacterial DBO molecules are referred to as “enhancers” of the activity of β-lactams that inhibit other PBPs besides PBP2, due to synergy arising during inhibition of multiple PBPs (25, 26). It is reasonable to suspect that the PBP2 variants such as those identified in this study may therefore also reduce this effect, but this remains to be determined. Nonetheless, the presence of these PBP2 variants in clinical isolates that also harbor the YRIN insertion in PBP3 (5) as well as *bla*_NDM-1_, provides yet another glimpse into the clinical emergence of multifactorial resistance, not only β-lactamase mediated but also involving multiple target mutations. The latter can clearly impact currently used therapeutics but may also hamper the future potential of novel β-lactam mimetics such as DBOs to the extent that their effectiveness relies on engaging these targets.
